# Whole-genome sequence of a female *Loa loa* adult worm from Cameroon

**DOI:** 10.1186/s13104-026-07775-w

**Published:** 2026-03-25

**Authors:** Jeanne A. Rajaonarivelo, Cédric Mariac, Philippe Cubry, François Sabot, Sébastien D. S. Pion

**Affiliations:** 1 TransVIHMI, University of Montpellier, INSERM, IRD, Montpellier, France; 2 DIADE, Univ Montpellier, CIRAD, IRD, Montpellier, France

**Keywords:** *Loa loa*, Oxford Nanopore Technologies, Whole-genome sequencing, Filarial nematode parasite

## Abstract

**Objective:**

Although loiasis affects more than 20 million people and that its associated clinical complications can cause multiple organ failures, this disease remains understudied. Consequently, the elimination of loiasis is not on the agenda of public health services. Defining an effective strategy to tackle this disease is challenging and requires a better understanding of the parasite’s biology, including its genetic aspects. *Loa loa* adult worms provide large amounts of biological tissues for genetic sequencing, but are very difficult to collect. Therefore, limited genomic data are available for this parasite. This study aimed to produce a more complete genome assembly of *L. loa* worm, which can be used for future research on genetic diversity, population dynamics and immunological processes.

**Data description:**

A 91.2 Mb genome assembly (150 contigs, N50 = 1.14 Mb, GC content = 30.67%) was generated from a single adult female *L. loa* worm, sequenced on a PromethION flow cell to a read depth of 147×. Alignment of this assembly with the available *Loa* reference genome displayed 96.08% identity. BUSCO analysis showed a 98.0% completeness of universally conserved nematode genes. The whole-genome assembly reported in this study were deposited in NCBI under the accession number JBSOQI010000000.

**Supplementary Information:**

The online version contains supplementary material available at 10.1186/s13104-026-07775-w.

## Objective

Loiasis is a disease endemic to West and Central Africa, caused by infection with the filarial worm *Loa loa*. Although loiasis affects more than 20 million people, this disease remains the least studied pathogenic filariasis as most of the cases seem asymptomatic [1]. However, there is growing evidence that clinical complications of loiasis are associated with multiple organ dysfunction, and may explain the excess mortality associated with high levels of *L. loa* infection [2]. Nevertheless, strategies for eliminating *L. loa* parasites remain challenging and require a deeper understanding of filarial biology [3]. Genomic approaches have provided insights into the genetic differentiation of filarial parasites and supported the development of diagnostic tools [4]. However, whole-genome sequencing of *L. loa* has constraints, and limited genomic data are available for this parasite [5]. Obtaining adult samples is complex (via surgical or optician procedures), and DNA extraction is often contaminated with human host DNA. The *L. loa* genome is estimated to be approximately 91.4 Mb, and consists of five autosomes and one sex chromosome [4, 6]. The first draft assembly, sequenced from microfilariae collected from a patient from Cameroon and generated using the Roche 454 FLX platform, was published in 2013 [6], with an N50 of 172 kb and 5,774 contigs. Then, in 2014, microfilarial genomes obtained from a blood sample of an infected individual in the Central African Republic were sequenced and bulk-assembled using reads generated by both Illumina and Pacific Biosciences platforms [5], producing a mixed assembly (N50 of 180 kb, 2,250 scaffolds).

Here, we produced the most complete, high-quality genome assembly of *L. loa* (see details in supplementary Tables [7, 8]), derived from a single adult female worm collected from a patient originating from Cameroon. We used Oxford Nanopore Technologies (ONT) sequencing to generate long reads that improve genome assembly contiguity and provide additional structural information [9]. This new improved assembly data can be used for future research on genetic diversity, population dynamics and immunological processes.

## Data description

The *L. loa* adult worm was collected during a surgery campaign conducted by the Cameroon-based Non-Governmental Organization Ascovime. The worm was discovered during an inguinal hernia surgery of a patient in Cameroon (Data File 1, Fig. 1; Table 1) [10]. Once the parasite had been removed, it was stored in a tube containing 70% ethanol until the DNA extraction in the laboratory. The adult worm was washed with Phosphate Buffered Saline (pH: 7.45), followed by nuclease-free water rinsing, then finely cut with a sterile scalpel and lysed at 56°C in 400 µl lysis buffer (50 mM Tris-HCl, pH 8.0; 100 mM NaCl; 20 mM EDTA; 1% SDS; Proteinase K, 2 mg/ml; RNase/DNase free H_2_O) until complete dissolution. We extracted DNA with potassium acetate (see details in Data File 2, Table 1) [11]. Library preparation for third-generation sequencing using an Oxford Nanopore Technology was performed using the Rapid Sequencing DNA V14 Barcoding Kit (SQK-RBK114.24, Oxford Nanopore Technologies, 2025) following the manufacturer’s recommendations. Seven indexed libraries were prepared using the same DNA, each from 200 ng of genomic DNA. Barcoded libraries were then pooled and cleaned up with AMPure XP magnetic beads (Beckman Coulter, USA). A total of 782.6 ng of DNA was loaded onto an ONT PromethION flow cell (FLO-PRO114M) for sequencing.

**Table 1:** Overview of data files/data sets

Label	Name of data file/data set	File types (file extension)	Data repository and identifier (DOI or accession number)
Data file 1	Figure 1. Overview of *L. loa* specimen and genomic representation	Image file (.jpg)	Figshare (10.6084/m9.figshare.30695753) [10]
Data file 2	Materials and Methods: DNA extraction, quantification, and quality assessment	Document file (.pdf)	Figshare (10.6084/m9.figshare.30695858) [11]
Data file 3	Raw sequencing data statistics	Excel (.xlsx)	Figshare (10.6084/m9.figshare.30706679) [18]
Data file 4	Genome assembly statistics and comparison with other published genomes	Excel (.xlsx)	Figshare (10.6084/m9.figshare.30708203) [7]
Data file 5	BUSCO results and comparison with other published genomes	Excel (.xlsx)	Figshare (10.6084/m9.figshare.31124986) [8]
Data set 1	*L. loa* genome assembly	Fasta file (.fasta)	NCBI (https://identifiers.org/ncbi/insdc.gca:GCA_053739935.1) [20]
Data set 2	*L. loa* raw sequencing data	Fastq file (.fastq)	NCBI (https://identifiers.org/ncbi/insdc.sra:SRP666436) [19]

ONT Pod5 were basecalled using the super-accuracy (SUP) model and demultiplexed with Dorado v1.0.1 (Oxford Nanopore Technologies Ltd). Adapter sequences and barcodes were trimmed using Porechop v0.2.4 (https://github.com/rrwick/Porechop). Quality of Nanopore sequencing data was assessed with NanoPlot v1.41.3 [12]. Raw reads were assembled using Raven-assembler v1.8.3 [13], then polished with Medaka v1.5 (https://github.com/nanoporetech/medaka) using the original ONT reads. Potential contaminants were identified using BlobTools v1.1.1 [14]. The genome assembly was aligned to the *L. loa* reference genome (GCA_039623295.1) using the web application D-Genies with Minimap2 v2.28 [15]. The assembly-stats v1.0.1 [16] was used to compute genome assembly statistics. Completeness of the genome assembly was evaluated with Busco v5.5.0 [17] using ‘nematoda_odb10’ as reference.

We sequenced a single adult female *L. loa* worm to a read depth of 147×. The PromethION flow cell generated 11,616,046 reads, with an N50 read length of 4,719 bases and a mean read length of 1,152 bases (Data File 3, Table 1) [18, 19]. The final assembled genome is 91.2 Mb in size, consists of 150 contigs with a contig N50 of 1.14 Mb and a GC content of 30.67% (Data File 4, Table 1) [7]. Alignment of the genome assembly with the *L. loa* reference genome (GCA_039623295.1) displayed 96.08% identity (Data File 1, Fig. 1; Table 1) [10]. Analysis with BlobTools identified no significant contamination, with most of the sequences assigned to nematodes (148 contigs, ~ 99% of the total span, 90.6 Mb), while the remaining two contigs (~ 0.6 Mb, < 1%) were unassigned taxonomically. The genome assembly contained 98.0% (3069/3131) of universally conserved genes in nematodes (BUSCOs), with a high proportion of single-copy genes 97.4% (3051), and a low percentage of duplicated (0.6%, 18), missing (1.1%, 34) and fragmented (0.9%, 28) genes (Data File 5, Table 1) [8]. The whole-genome assembly reported in this study were deposited in NCBI, under the accession number JBSOQI000000000 (Data set 1, Table 1) [20].

## Limitations

The assembled genome has not been annotated, and gene prediction is needed for future work to enable comprehensive comparative genomics. However, it is important to note that our assembly is derived from a single individual parasite and not from a pool of parasites, which limits potential assembly artefacts [21].

## Supplementary Information

Supplementary Material 1.

Supplementary Material 2.

Supplementary Material 3.

Supplementary Material 4.

Supplementary Material 5.

## Data Availability

The whole-genome assembly reported in this study was deposited in NCBI, under the accession number JBSOQI000000000 and BioProject ID PRJNA1347375. The data files and datasets generated in this study have been assigned unique identifiers, which are presented in Table 1 and referenced [7, 8, 10, 11, 18, 19, 20].

## Abbreviations

BUSCOs: Benchmarking Universal Single-Copy Orthologs
D-Genies: Dot plot large Genomes in an Interactive, Efficient and Simple way
DNA: Deoxyribonucleic Acid
GC content: Guanine-Cytosine content
NCBI: National Center for Biotechnology Information
ONT: Oxford Nanopore Technologies
SDS: Sodium Dodecyl Sulfate
