# Supplementary figures and images for: Whole-genome sequence of a female Loa loa adult worm from Cameroon
Source: BMC Res Notes. 2026 Mar 25;19:201. doi: 10.1186/s13104-026-07775-w (PMC13137628; doi:10.1186/s13104-026-07775-w)

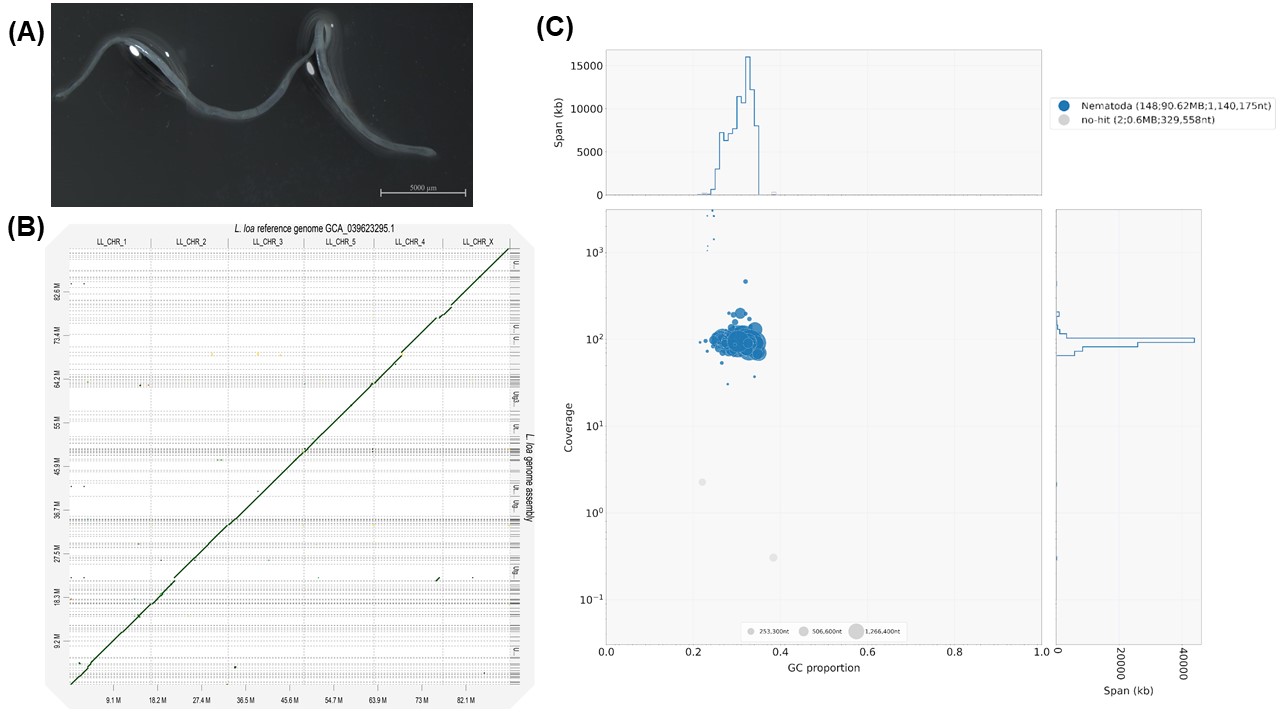

Supplement: Supplementary file 1 — Supplementary Material 1. [file 13104_2026_7775_MOESM1_ESM.jpg]
