# Supplementary material for: Whole-genome sequence of a female Loa loa adult worm from Cameroon
Source: BMC Res Notes. 2026 Mar 25;19:201. doi: 10.1186/s13104-026-07775-w (PMC13137628; doi:10.1186/s13104-026-07775-w)
Supplement: Supplementary file 2 — Supplementary Material 2. [file 13104_2026_7775_MOESM2_ESM.pdf]

Materials and Methods: DNA extraction, quantification, and quality assessment

Worm was lysed at 56 °C with gentle agitation (300 rpm) in 400 µl lysis buffer (50 mM Tris-HCl, pH 8.0; 100 mM NaCl; 20 mM EDTA; 1% SDS; Proteinase K, 2 mg/ml; RNase/DNase free H_2O) until complete dissolution. Protein precipitation was performed by adding 200 µl of 3 M potassium acetate, followed by incubation on ice for 30 min. The mixture was centrifuged at 14,000 rpm for 10 min at 4 °C, and the clarified supernatant was transferred to a new tube. Then DNA was precipitated in 0.6 volume of cold isopropanol and 0.1 volume of sodium acetate (3M pH=5), which were then incubated during one night at -20°C. After centrifugation at 14,000 rpm for 30 min at 4°C, supernatant was removed. The DNA pellet was washed with 1 ml ethanol 70%, followed by centrifugation at 14,000 rpm for 10 min at 4°C. DNA pellet was air dried at room temperature and resuspended with 50 µl of Tris-EDTA buffer, and stored at -20°C prior to use.

Concentration of extracted DNA was measured with a Qubit Flex Fluorometer using the 1x dsDNA HS assay kit (Invitrogen, Thermo Fisher Scientific, France) and the purity was assessed with spectrophotometric measurement using a Nanodrop One (Thermo Fisher Scientific, France), for estimating the A_{260}/A_{280} and A_{260}/A_{230} absorbance ratios. The size of DNA fragments was then evaluated by electrophoresis on a 2% agarose gel prepared in 1× TAE buffer, which was run during 35 min at 135 V.
